# Biomimetic polymersomal nanoreactors for hepatocyte-directed detoxification in drug-induced liver injury

**DOI:** 10.1016/j.mtbio.2025.102169

**Published:** 2025-08-05

**Authors:** Wenxing Gu, Ruxue Bai, Jiaxin Wang, Jun Wang, Chongzhou Fang, Yuanchao Shi, Peixing Wang, Qiaoqiao Wang, Wei Bing, Tian Xie, Jing Mu

**Affiliations:** a Institute of Precision Medicine, Peking University Shenzhen Hospital, Shenzhen, 518036, China; b School of Pharmacy, Zhejiang Provincial Key Laboratory of Anti-Cancer Chinese Medicines and Natural Medicines, Hangzhou Normal University, Hangzhou, 311121, China; c School of Chemistry and Life Science, Changchun University of Technology, Changchun, 130012, China; d Key Laboratory of Bionic Engineering, Ministry of Education, Jilin University, Changchun, 130022, China

**Keywords:** Hepatocyte targeting, Polymersome, Nanoreactor, Drug-induced liver injury, Reactive oxygen species

## Abstract

Drug-induced liver injury (DILI) is a major clinical concern associated with drug toxicity, primarily characterized by hepatocyte damage driven by oxidative stress and inflammation. Excessive accumulation of reactive oxygen species (ROS) and pro-inflammatory cytokines exacerbates liver injury, necessitating effective therapeutic interventions. While natural antioxidant enzymes exhibit high catalytic activity and specificity in ROS scavenging, their clinical use is limited by poor stability in circulation and limited cellular uptake. To address these challenges, we developed antioxidant polymersomal nanoreactors (CS@PS) for the targeted co-delivery of catalase (CAT) and superoxide dismutase (SOD). CS@PS functions as a cascade system, efficiently neutralizing ROS and mitigating inflammation in hepatocytes. Notably, systemic administration of CS@PS exhibits targeted delivery to hepatocytes, ensuring enzyme stability and bioavailability. In a model of acetaminophen-induced liver injury, CS@PS significantly reduced hepatic ROS levels and alleviated liver damage, underscoring its therapeutic potential. This study highlights the promise of CS@PS as an innovative antioxidant nanoreactor for DILI treatment offer a promising strategy for managing other ROS-mediated diseases.

## Graphical abstract

The antioxidant biomimetic polymersomal nanoreactors (CS@PS) were developed for the hepatocyte targeted co-delivery of catalase and superoxide dismutase, efficiently neutralizing ROS and mitigating inflammation in drug-induced liver injury.

**Figure:**
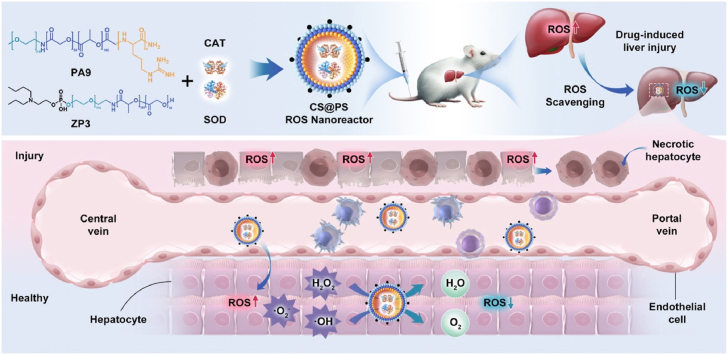

## 1 Introduction

Drug delivery systems (DDS) have significantly improved the efficacy and safety of therapeutic agents by optimizing their pharmacokinetics and biodistribution [[1], [2], [3], [4]]. However, clinical translation remains challenging due to systemic toxicity and low bioavailability, which often contribute to treatment failure [5]. Recently, organ-targeting DDSs, which concentrates therapeutic agents directly at the site of interest, have emerged as a promising strategy to achieve higher local drug concentrations, improved pharmacokinetics, and enhanced biodistribution profiles [6,7]. Such targeted approaches are particularly valuable for treating diseases that are localized to specific organs, thereby maximizing the drug efficacy and minimizing off-target toxicity [8]. Nowadays, organ-selective vectors have shown broad applications in the delivery of proteins, small interfering RNA (siRNA), and messenger RNA (mRNA) [9,10]. For instance, lung-targeted LNPs have been developed for precise homology-directed repair-mediated CRISPR/Cas genome correction in cystic fibrosis mouse models [11]. Spleen-targeted lipid nanoparticles (LNPs) that encapsulate mRNA encoding the chimeric antigen receptor (CAR) gene have been utilized for *in situ* CAR T cell generation, significantly extending median survival in murine models of lymphoblastic B-cell lymphoma [12]. Additionally, we have previously established a library of polymersomes designed for selective spleen and liver targeting, and successfully applied a spleen-targeted DDS for antigen protein delivery in oncology vaccine development [13].

Building upon the success of organ-specific delivery strategies, particular attention has turned to the liver—a central organ in drug metabolism and a frequent site of toxicity—making it a particularly critical target for advanced DDS development. As the central hub of metabolic activity and xenobiotic processing, the liver is highly susceptible to damage from various endogenous and exogenous compounds [[14], [15], [16]]. Dysfunction of the liver can lead to various metabolic disorders and is implicated in a broad spectrum of diseases, including fatty liver, hepatitis, cirrhosis, and hepatocellular carcinoma [[17], [18], [19]]. Notably, drug-induced liver injury (DILI) is a significant clinical concern, arising from direct or indirect hepatic damage caused by drugs or their metabolites during medication [20,21]. This is primarily due to the liver's critical function in metabolizing xenobiotics that enter the gastrointestinal tract [22,23]. Furthermore, DILI is a major factor limiting drug prescriptions and frequently leads to the discontinuation of clinical development programs for candidates [24]. DILI can occur in response to specific therapeutic agents, such as anti-tuberculosis and anticancer drugs, as well as widely used medications like acetaminophen and amoxicillin, both of which have been implicated in severe liver injury [25]. In this context, the development of liver-targeted DDSs is imperative—not only to enhance the therapeutic efficacy against liver-specific diseases, but also to mitigate hepatotoxic effects, thereby improving drug safety and expanding the clinical utility of a wide range of therapeutics.

Extensive studies have identified oxidative stress driven by the excessive production of reactive oxygen species (ROS) are a key mechanism in DILI [[26], [27], [28]]. The oxidative imbalance leads to damage to cellular components, disruption of mitochondrial homeostasis, and activation of pro-inflammatory signaling pathways [29,30]. Damaged mitochondria produce an excessive amount of ROS, particularly superoxide anion radical (O_2_^•−^), hydroxyl radical (•OH), and hydrogen peroxide (H_2_O_2_) [31]. While endogenous antioxidant enzymes such as superoxide dismutase (SOD), catalase (CAT), and peroxidase (POD) play critical roles in ROS detoxification, their capacity is often insufficient to counteract the excessive oxidative burden in DILI [32]. Although Broad-spectrum antioxidants, such as N-acetylcysteine have been explored for ROS scavenging, their low bioavailability and lack of target specificity: limit their effectiveness in DILI treatment [33]. Given the complex and multifactorial nature of DILI, a more effective strategy involves the simultaneous removal of multiple ROS species to comprehensively mitigate liver damage [[34], [35], [36]]. Therefore, the development of hepatocyte-targeted, multiple ROS-scavenging reactors with stable and efficient delivery systems is highly required.

**Scheme 1:**
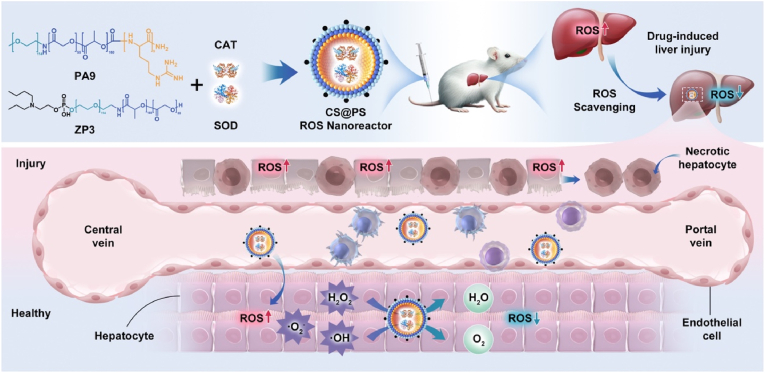
Schematic illustration of the design of hepatocyte-targeted antioxidant polymersomal nanoreactors (CS@PS) for treatment of drug-induced liver injury.

In this work, we rationally design a hepatocyte-targeted biomimetic cascade system (CS@PS) for the efficient depletion of multiple ROS, including O_2_^•−^ and H_2_O_2_, to enhance the treatment of DILI (Scheme 1). The system integrates the natural enzymes catalase (CAT) and superoxide dismutase (SOD) into a combinatorial polymeric framework composed of poly(ethylene glycol)-block-poly(lactide-co-glycolic acid) (PEG-PLGA) functionalized with the oligopeptides PEG-PLGA-OA9 (polymer PA9) and ZP3-PEG-PLGA (polymer ZP3). During the self-assembly in aqueous condition, the positive charged guanidinium groups of PA9 facilitate the encapsulation of CAT and SOD within the aqueous interiors, while the amphipathic ions in ZP3 exposure on the coronas, modulating the hepatocyte targetability. CS@PS features a semi-permeable membrane that allows the selective passage of small ROS molecules while protecting CAT and SOD from enzymatic degradation. By effectively scavenging the extracellular and intracellular ROS within hepatocyte tissues, CS@PS mitigates oxidative damage and suppresses inflammation, thereby offering significant hepatoprotective effects. The innovative strategy presents a promising platform for combating oxidative stress-related liver diseases, addressing a critical unmet need in hepatology.

## 2 Results and discussion

### 2.1 Preparation and characterization of CS@PS

**Fig. 1:**
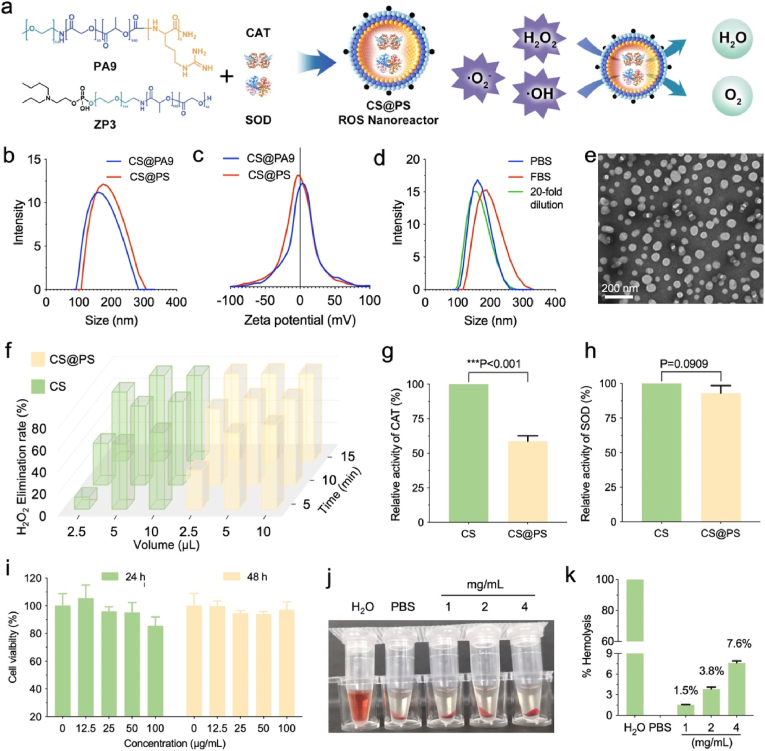
a) The schematic illustration of the nanoreactor CS@PS. b) Hydrodynamic diameter distribution of the CS@PS. c) Zeta-potential of the CS@PS. d) Colloidal stability of the CS@PS in serum for over two weeks. e) TEM image of the CS@PS. f) H_2_O_2_ elimination rate of the CS@PS at different incubation conditions, CS (CAT and SOD) as the control. g-h) The relative activity of CAT and SOD in CS@PS compared to CS. i) The viability of FL83B cells treated with CS@PS under different treatment conditions. j-k) Hemolysis analysis of RBCs treated with CS@PS at the concentration of 1, 2 and 4 mg mL^−1^, where water and PBS were used as the positive/negative controls.

To obtain the antioxidant polymersomal nanoreactors (CS@PS), two polymers, namely, PA9 and ZP3, were synthesized (Figure S1-S3, Supporting Information). The positively charged guanidinium groups in PA9 facilitated the encapsulation of the antioxidant enzymes, while the amphipathic ions in ZP3 played a crucial role in modulating the organ-specific targeting of the nanoreactors (Fig. 1a). CS@PS was prepared using a self-assembly strategy, a mild and straightforward process optimized to preserve the bioactivity of the encapsulated enzymes. Dynamic light scattering (DLS, Fig. 1b and c) analysis revealed that the average size of CS@PS was approximately 150 nm with a narrow polydispersity index (PDI = 0.2). The neutral surface zeta potential and PEGylation of CS@PS enables reduced non-specific protein adsorption and prolonged circulation time. To assess the role of amphipathic ions in modulating targeting capability, CS@PA9 as a control was obtained by loading CAT and SOD using PA9 only. The particle size and surface zeta potential of CS@PA9 were similar to those of CS@PS. In order to evaluate the stability in blood circulation, CS@PS was incubated in simulated biological buffers (10 % bovine serum albumin containing PBS) at 37 °C (Fig. 1d). Negligible changes in particle size were observed during 48-h incubation, suggesting potential favored stability in physiological conditions. Furthermore, transmission electron microscopy (TEM) characterization (Fig. 1e) depicted CS@PS particles are uniform in size with a spherical morphology, clearly validating the successful preparation of the designed polymersomal nanoreactors.

Subsequently, the catalase (CAT) and superoxide dismutase (SOD) enzyme activities of CS@PS were examined *via* the addition of substrates H_2_O_2_ and superoxide dismutase assay kit, respectively. As shown in Fig. 1f–g and Fig. S4 (Supporting Information), the concentration of H_2_O_2_ gradually decreased over time and the addition of CS@PS notably accelerated the degradation of H_2_O_2_. A similar trend was observed in the SOD enzyme activity assay, confirming the catalytic efficiency of CS@PS in ROS scavenging (Fig. 1h and Fig. S5-S6, Supporting Information). The CAT/SOD enzyme activity results, presented as heatmaps, revealed that CS@PS retained approximately 60 % and 90 % in the relative activity of CAT and SOD respectively, compared to the free CAT/SOD. The high enzyme activities indicate that the membrane of the polymersome is permeable to small molecules such as H_2_O_2_ and O_2_^•−^. Moreover, the impermeability to macromolecules could effectively prevent the encapsulated enzymes from degradation in biological context with various proteases or other enzymes. Therefore, CS@PS could be considered as a biocatalytic nanoreactor, wherein the encapsulated CAT/SOD enzymes transform ROS into O_2_ and H_2_O. Before evaluating its cellular catalytic performance, we next investigated the biocompatibility of CS@PS by CCK-8 assay and hemolysis test. *In vitro* cytotoxicity (Fig. 1i) showed that CS@PS exhibited no significant toxicity toward the hepatocyte cell line (FL83B), even at a high concentration of 100 μg/mL. In addition, CS@PS caused minimal hemolysis (3.8 %) to RBCs at concentrations up to 2 mg/mL (Fig. 1j and k). These findings highlight the polymersomes CS@PS as an effective and biocompatible carrier for the delivery of CAT/SOD enzymes.

### 2.2 *In vitro* evaluation of CS@PS

**Fig. 2:**
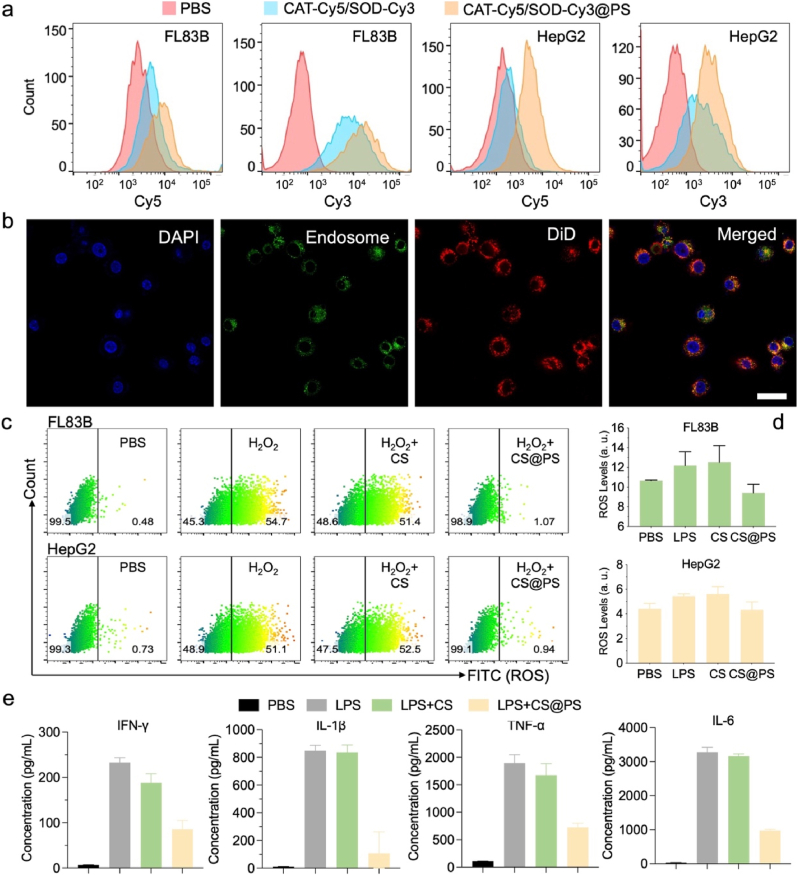
a) Cellular uptake efficiency analysis by flow cytometry in FL83B and HepG2 cells treated with free CAT-Cy5 and SOD-Cy3, or CAT-Cy5/SOD-Cy3@PS for 4 h incubation. b) Endosomal escape of DiD-doped CS@PS-DiD in FL83B cells observed by confocal microscopy with 6 h incubation. The nuclei and endo/lysosomes were stained with Hoechst (blue) and Lysotracker (green), respectively. DiD was colored in red. Scale bars, 20 μm. c) ROS levels in untreated and CS@PS-treated FL83B and HepG2 cells incubated with 100 μM H_2_O_2_. d) ROS Fluorescence quantitative analysis in untreated, CS (CAT and SOD) and CS@PS-treated FL83B and HepG2 cells stimulated with LPS. e) The inflammatory factors analysis in FL83B cells with different treatments.

To investigate the *in vitro* cellular uptake efficiency of CS@PS, Cy5-labeled CAT and Cy3-labeled SOD were loaded into the polymersomes. The uptake of CS@PS was evaluated by flow cytometry analysis (Fig. 2a and Fig. S7, Supporting Information). In the absence of polymersomes carriers, free CAT/SOD exhibited limited uptake in FL83B and HepG2 cells. Notably, the cellular uptake was significantly increased by 3-5-fold after CAT and SOD were loaded into polymersomes. These results verified that polymersome carriers greatly enhanced the intracellular delivery of CAT and SOD. Additionally, cellular distribution experiments were performed with CS@PS-DiD. As illustrated in Fig. 2b, most of the CS@PS-DiD were observed to be distributed outside the lyso/endosomes after 6 h, which indicated that the guanidinium groups-containing polymersomes facilitated the efficient endosomal escape of CS@PS, further enhancing intracellular enzyme availability.

CS@PS-mediated ROS scavenging efficacy was subsequently assessed in FL83B and HepG2 cells. 2′,7′-dichlorofluorescein diacetate (DCFH-DA) fluorescent probe was applied as the ROS indicator. As shown in Fig. 2c, H_2_O_2_-treated cells exhibited high ROS levels as compared to the control cell group without any treatment. The pretreatment of free CS resulted in negligible changes in ROS levels. By contrast, when treated with CS@PS, the intracellular ROS levels was remarkably decreased, highlighting the superior ROS scavenging ability of CS@PS. Additionally, the anti-inflammatory capacity of CS@PS was evaluated with ROS levels and inflammatory cytokines. Lipopolysaccharide (LPS)-treated FL83B and HepG2 cells showed a significant decrease in ROS levels upon the addition of CS@PS, bringing them close to the baseline intracellular ROS level (Fig. 2d and Figure S8-S9, Supporting Information). Besides, the inflammatory cytokines levels, including INF-γ, IL-6, IL-1*β*, and TNF-*α*, in CS@PS-treated cells were also significantly reduced, while CS-treated group showed negligible effects (Fig. 2e). These findings collectively demonstrated that CS@PS exhibited remarkable intracellular anti-inflammatory efficacy.

### 2.3 *Ex vivo* imaging hepatocyte-targeted accumulation of CS@PS

**Fig. 3:**
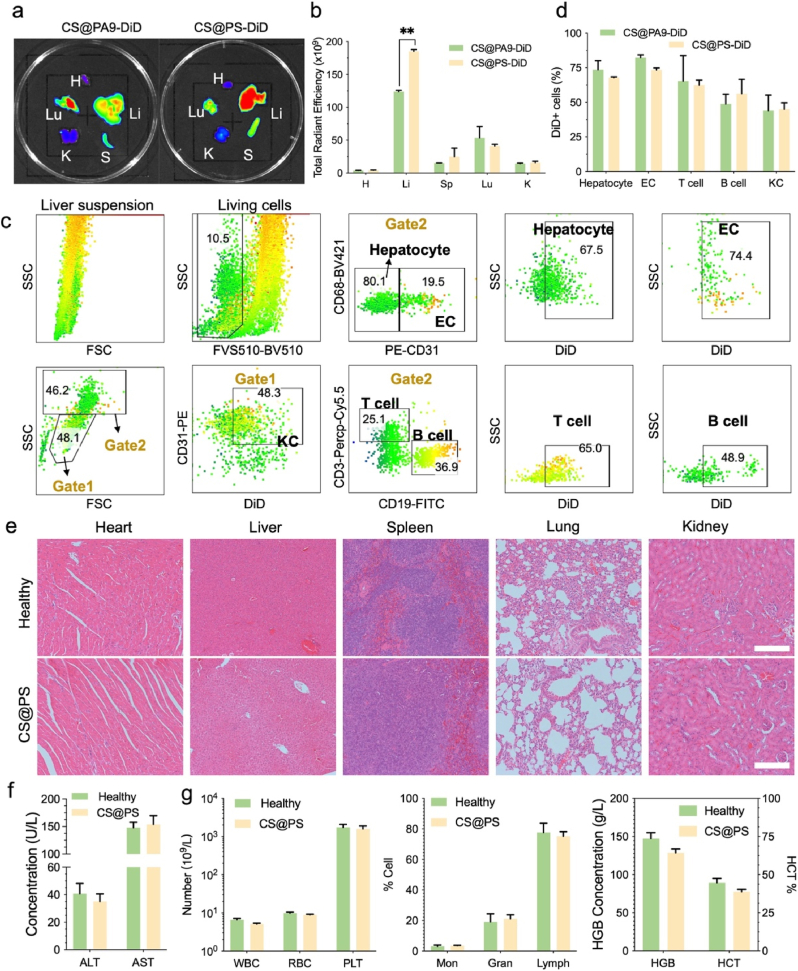
a–b) NIR fluorescence images and quantitative analysis of major organs at 6 h post intravenous injection of DiD dye-doped CS@PS-DiD. Non-hepatocyte-specific CS@PA9-DiD as the control. c) Flow cytometry analysis for liver cell fractions in in vivo experiments. d) Analysis of DiD + cells extracted from liver of mice treated with CS@PS-DiD and CS@PA9-DiD. e) H&E staining images of main organs from mice received CS@PS. Scale bar: 100 μm. f-g) The blood biochemistry and blood routine analysis of mice received CS@PS. Healthy mice as the control.

For liver-targeted imaging purpose, CS@PS or CS@PA9 were labeled with fluorescent dye DiD (2 % *w/w*) during their self-assembly in aqueous condition, affording monodispersed nanoparticles termed as CS@PS-DiD or CS@PA9-DiD, respectively. As shown in Fig. 3a and b, CS@PS-DiD exhibited significantly higher accumulation in the liver, while the non-targeted CS@PA9-DiD was distributed in both in liver and lung. The analysis of CS@PS-DiD endocytosis into different cells of the liver revealed that CS@PS-DiD was endocytosed into hepatocytes, endothelial cells (EC), T cells, B cells, and Kupffer cells (KC), with hepatocytes and EC exhibiting the highest uptake rates, being approximately 75 % (Fig. 3c and d). These results confirmed that CS@PS could effectively deliver CAT and SOD to the majority of hepatocytes and EC, the key cellular players in ROS-induced liver injury, thereby enhancing its therapeutic potential for liver protection.

Prior to the initiation of treatment experiments in the animal model, an assessment was conducted to ascertain the safety of CS@PS. After administration of CS@PS into mice, the major organs were harvested and subjected to histological examination using the hematoxylin and eosin (H&E) staining technique. As illustrated in Fig. 3e, minimal damage was observed in major organs including the heart, liver, spleen, lungs, and kidneys. Furthermore, we collected whole blood and serum from mice to assess blood biochemistry and blood routine. The results of these tests indicated that CS@PS exhibited no apparent toxicity, confirming its compliance with safety requirements. (Fig. 3f–g and Fig. S10).

### 2.4 *In vivo* therapeutic effect of CS@PS against DILI

**Fig. 4:**
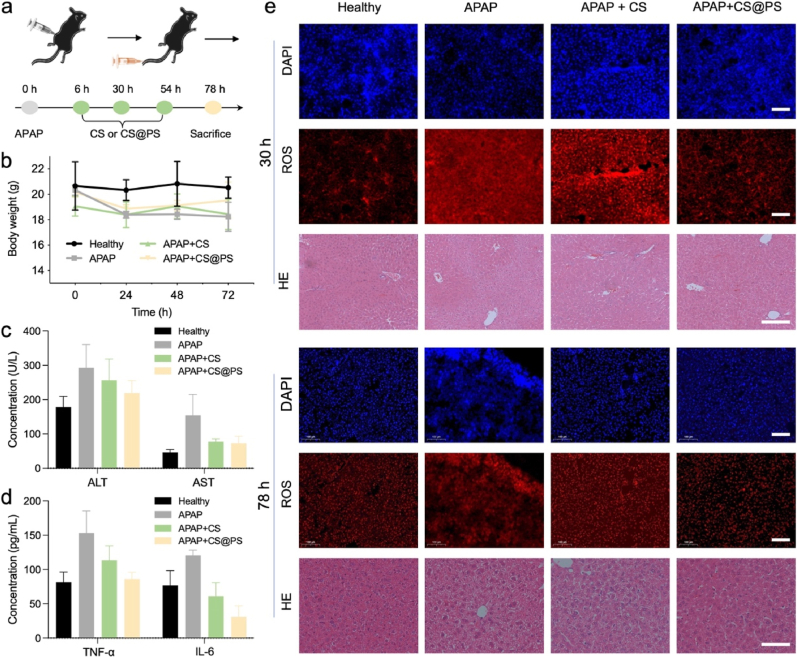
a) Timeline of the DILI mice model establishment and treatment. b) Body weight profile of each indicated treatment group (n = 10 mice/group). c) Serum aminotransferase levels (AST and ALT) of mice treated with CS or CS@PS at 30 h (n = 3 mice/group). d) Serum inflammatory factors (TNF-α and IL-6) of mice treated with CS or CS@PS at 30 h (n = 3 mice/group). e) *Ex vivo* FL analysis of ROS levels and histological images in the liver tissues of mice treated with CS or CS@PS at 30 and 78 h. Scale bar: 100 μm. Data represent means ± SD.

In order to investigate the therapeutic efficacy, a DILI mice model was established by intraperitoneal injection of acetaminophen (APAP, Fig. 4a). At 6, 30 and 54 h after APAP injection, CS@PS was administered *via* the tail vein to the model mice. The control groups included the healthy group, APAP group (untreated group) and APAP + CS group (CS treatment group). During the experimental period, the body weights of the mice in all groups were monitored (Fig. 4b). It is noteworthy that the body weight of the mice in the APAP group exhibited a continuous decline (ca. 10 % weight loss), revealing severe DILI. CS or CS@PS treatment did not prevent the initial weight loss within the first 24 h, suggesting the acute toxicity of high-dose APAP. However, in the following days, body weights in the CS@PS group showed a significant recovery, demonstrating that CS@PS effectively alleviated DILI and promoted recovery.

The serum aminotransferase levels, including aspartate aminotransferase (AST) and alanine aminotransferase (ALT), were employed as indicators of liver function and severity of DILI. As illustrated in Fig. 4c and Fig. S11, APAP-treated mice exhibited significant elevation in ALT and AST levels at 30 h post-injection, thereby validating the succeed establishment of DILI mice model. Notably, treatment with CS@PS effectively restored ALT and AST levels to those observed in healthy mice, indicating the excellent protective effect against DILI. Although CS offered certain degree of protection, its efficacy was inferior to that of CS@PS. To further investigate the anti-inflammatory effects of CS@PS, the levels of inflammatory cytokines in serum were evaluated (Fig. 4d and Fig. S12, Supporting Information). As shown in Fig. 4d, the levels of TNF-α and IL-6 were remarkably increased in APAP-treated mice group. However, CS@PS treatment significantly suppressed the expression of TNF-α and IL-6, mitigating inflammatory damage. These findings highlight the potent anti-inflammatory and hepatoprotective properties of CS@PS in alleviating APAP-induced liver injury.

Subsequently, the accumulation of ROS in the liver tissue further assessed by dihydroethidium staining. As illustrated in Fig. 4e, the ROS level in the APAP-treated group was significantly elevated in comparison to healthy mice at both 30 and 78 h. In contrast, mice treated with CS@PS showed a pronounced reduction in ROS levels, indicating CS@PS effectively alleviated the liver injury by scavenging excessive ROS. Moreover, the persistently high ROS levels in the CS treatment group highlighted the crucial role of liver-targeted delivery in enhancing therapeutic efficacy. Besides, the histological examination was performed to evaluate liver tissue damage. As expected, the APAP group revealed significant liver damage, characterized by extensive hepatocyte degeneration and necrosis, and the formation of an empty reticular fiber scaffold due to necrotic cell loss.

**Fig. 5:**
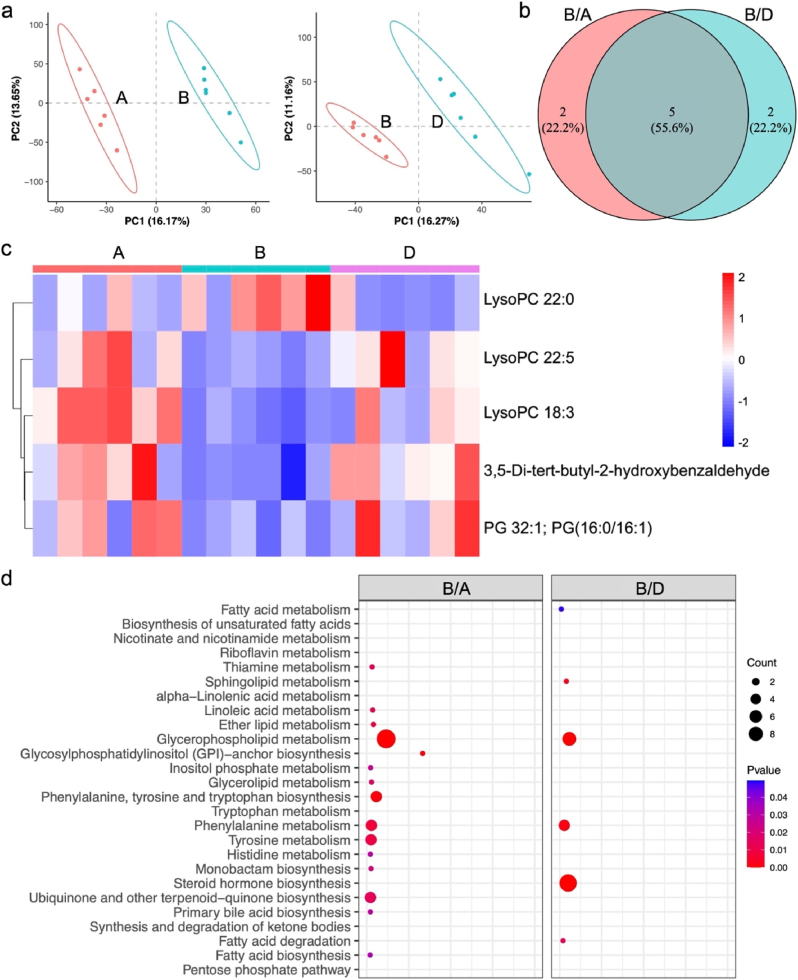
a) The PLSDA score plot in B/A and B/D comparisons. b) Venn diagram of the DEMs identified from pairwise comparisons. c) Thermographic analysis of potential biomarkers in the secondary identification of metabolite in A/B/D comparisons. d) The KEGG pathways associated with the DEGs identified in B/A and B/D comparisons, respectively.

Finally, untargeted metabolomics was employed to elucidate metabolic alterations in response to APAP exposure and CS@PS treatment. Mice were categorized into groups: A: Healthy mice, B: APAP group, D: APAP + CS@PS group. The Partial Least Squares Discriminant Analysis (PLS-DA) showed that all quality control (QC) samples were tightly clustered, indicating that the metabolomics analysis exhibited good stability and repeatability (Fig. 5a, B/A and D/B). The fitting check of the PLS-DA model demonstrated that the intercept of Q2 was −0.5268, −0.5301, and −0.4639 respectively, indicating no risk of overfitting (Figure S13-S14, Supporting Information). Differential analysis identified 112 differentially expressed metabolites (DEMs) between the B/A groups, with 5 DEMs across comparisons in the B/A and D/B groups, confirming that both APAP exposure and CS@PS treatment led to significant shifts in liver metabolism (Fig. 5b). Notably, specific DEMs-LysoPC(22:0), LysoPC(22:5), LysoPC(18:3), 3,5-di-tert-butyl-2-hydroxybenzaldehyde, PG(32:1); PG(16:0/16:1)—were significantly dysregulated in the APAP group and restored to near-normal levels following CS@PS treatment. This pattern was clearly reflected in the hierarchical clustering heatmap (Fig. 5c, Figures S15–S16), suggesting that CS@PS effectively reversed the metabolic disruptions induced by APAP.

These metabolites provide mechanistic insights into the protective effects of CS@PS. For example, LysoPCs and PGs are crucial components of membrane lipid metabolism and cellular signaling. These metabolites provide mechanistic insights into the protective effects of CS@PS. For example, LysoPCs and PGs are crucial components of membrane lipid metabolism and cellular signaling. The normalization of LysoPC and PG species strongly suggests restoration of membrane homeostasis and mitochondrial function, likely through the catalytic detoxification of ROS *via* its encapsulated CAT and SOD enzymes. Moreover, 3,5-di-tert-butyl-2-hydroxybenzaldehyde, a known antioxidant, was recovered in CS@PS group, supporting its role in reducing oxidative stress [37]. Importantly, Kyoto Encyclopedia of Genes and Genomes (KEGG) pathway enrichment analysis revealed that the DEMs were primarily involved in tryptophan/tyrosine metabolism and steroid hormone/ubiquinone biosynthesis (Fig. 5d). Disruption of tryptophan and tyrosine metabolism is known to be linked with inflammatory responses and oxidative stress [38], both central features of APAP-induced hepatotoxicity. Recovery of these pathways may reflect the ability of CS@PS to rebalance amino acid metabolism and attenuate inflammatory signaling. Furthermore, modulation of steroid hormone and ubiquinone biosynthesis pathways implies an impact on mitochondrial function and redox homeostasis, consistent with our proposed mechanism involving ROS clearance and anti-inflammation [39]. Taken together, the metabolomic findings demonstrate that CS@PS treatment effectively reverses key APAP-induced metabolic disruptions, particularly in lipid metabolism, redox balance, and amino acid pathways. The recovery of specific DEMs to control levels, along with enrichment of relevant protective pathways, underscores the systemic efficacy of CS@PS in mitigating drug-induced liver injury.

## 3 Conclusions

The development of drug delivery systems (DDS) aims to address the inherent limitations of conventional therapies. Despite their promising advantages, DDS still face significant challenges in clinical translation, including suboptimal therapeutic efficacy and potential toxicity. The emerging organ-targeted strategies have demonstrated considerable promise, particularly in the treatment of liver-associated disorders. Drug-induced liver injury (DILI) is one of the most common and serious adverse drug reactions, leading to acute liver failure or even death in severe cases. The objective of this study was to develop a hepatocyte-targeted DDS for improving the treatment of DILI. We optimized polymersome-based nanoreactor for the co-delivery of catalase (CAT) and superoxide dismutase (SOD) *via* a mild self-assembly strategy. The semi-permeable membrane of nanoreactor allowed selective passage of small ROS molecules while shielding CAT/SOD from enzymatic hydrolysis. In the acetaminophen-induced liver injury mouse model, CS@PS exhibited excellent anti-cellular ROS and anti-inflammatory abilities, which significantly ameliorated the liver injury. In addition, the untargeted metabolomics identified that tryptophan and tyrosine metabolism played a key role in CS@PS-mediated hepatoprotection.

Currently, the standard treatment for APAP overdose -NAC therapy is limited by a narrow therapeutic window, and a subset of patients fails to respond adequately. Although nanoparticle-based therapies, such as nanozymes, have shown promise in DILI treatment by mimicking the enzymatic activities of SOD/CAT, their catalytic efficiency is often suboptimal, and their *in vivo* safety profiles remain a concern, particularly for inorganic nanoparticles. The innovative design CS@PS incorporates a semi-permeable membrane that facilitates the selective permeation of small ROS molecules while protecting CAT and SOD from enzymatic degradation, thereby enhancing both catalytic activity and bioavailability. CS@PS can be used as a complementary therapy and even be used with NAC to broaden the treatment window and reduce dose-dependent toxicity. However, to facilitate clinical translation, several challenges must be addressed. These include comprehensive evaluations of long-term biosafety, immunogenicity, large-scale manufacturing consistency, and regulatory approval pathways. Future studies should also explore the therapeutic efficacy of CS@PS in diverse clinical contexts of ROS-mediated liver injury, such as ischemia-reperfusion and chronic hepatitis. Given its robust antioxidative properties, CS@PS holds great potential as a general therapeutic agent for ROS-mediated disorders. Furthermore, this liver-targeted polymersome may serve as a versatile platform for liver-specific drug delivery, paving the way for broader applications in hepatology and precision medicine.

## 4 Experimental

### 4.1 Materials

PEG-PLGA (molecular weight of PEG, PGA, and PLA: 5000, 3200, and 11500 Da, respectively) and PLGA (PGA/PLA (75/25), molecular weight ∼15000 Da) were purchased from Daigang Biomaterial, Jinan, China. Oligo-arginine (OA9, sequence: NH_2_-RRRRRRRRR-Ac) was purchased from China Peptides Co., Ltd, 98 %, Shanghai, China. HO-PEG-NH-BOC was purchased from Ponsure Biotechnology, Shanghai, China. Lipopolysaccharide (LPS), catalase (CAT, 20 kU/mg proteins) and superoxide dismutase (SOD, 3 kU/mg proteins) were purchased from Sigma-Aldrich, Shanghai, China. FITC anti-mouse CD19 (clone: 1D3, BD Pharmingen), PE anti-mouse TCR β (clone: H57-597, BD Pharmingen), PC7 anti-mouse CD11b (BD Pharmingen) PE anti-mouse CD31 (clone: MEC 13.3, BD Pharmingen). BV421 anti-mouse CD68 (clone: FA/11, BD Pharmingen), PerCP-Cy5.5 anti-mouse CD3e (clone: 145-2C11, BD Pharmingen) were purchased from Univision Bioscience Co., Ltd, Shanghai, China.

### 4.2 Synthesis

Polymers PA9 (PEG-PLGA-OA9) and ZP3 (ZP-PEG-PLGA) were synthesized in accordance with the previously described synthetic method. To obtain PA9, the hydroxyl group in PEG-PLGA was firstly activated by p-NPC and then reacted with the amino group in oligo-arginine (OA9). To obtain ZP3, HO-PEG-NH-BOC was firstly reacted with 2-Chloro-2-oxo-1,3,2-dioxaphospholane and deprotected the amino groups to obtain ZP3-PEG-NH_2_, following reacted with Poly (Lactic-Co-Glycolic acid)-N-Hydroxysuccinimidyl ester (PLGA-NHS) to obtain polymer ZP3.

### 4.3 Preparation and characterization of antioxidant polymersomal nanoreactors

The polymers (2000 μg, 20 mg/mL, DMSO) were slowly dropped into PBS buffer (1 mL, 10 mM, pH 7.4) containing CAT (25 μg, 500 U) and SOD (175 μg, 375 U). After stirring for 30 min, free CAT and SOD were removed by dialysis in PBS buffer (10 mM, pH 7.4) with a molecular weight cutoff of 300 kDa to obtain antioxidant polymersomal nanoreactors CS@PS. The theoretical loading content was set as 10 %, and calculated according to the following formula: theoretical loading content (wt.%) = (weight of enzymes in feed/weight of polymers) × 100. The mean hydrodynamic diameter, polydispersity index (PDI) and zeta potential were determined using DLS.

### 4.4 CAT and SOD enzymatic activity of CS@PS

The enzymatic activity of CAT was measured with CAT assay kit (Beyotime, Shanghai, China) according to the protocols: 100 μmol/L of hydrogen peroxide solution was added into a 96-well plate, and then different concentrations of CS@PS were added. After incubation at 37 °C for different times, 100 μl of hydrogen peroxide assay reagent was added to each well. Following gentle shaking or tapping at room temperature for 30 min, and the reaction was immediately analyzed using a microplate reader at absorbance wavelength of 560 nm. The concentrations of hydrogen peroxide in the samples were calculated from the standard curve. The enzymatic activity of SOD was measured with SOD assay kit (Beyotime, Shanghai, China) according to the following protocols: The 96-well plates were filled with different concentrations of samples, SOD assay buffer and WST-8/enzyme working solution sequentially, and the reaction initiation working solution was added and mixed thoroughly. After incubation at 37 °C for 30 min, and reaction was immediately analyzed using a microplate reader at absorbance wavelength of 450 nm. The CAT and SOD enzymatic activity of CS@PS were calculated using free CAT and SOD as the controls.

### 4.5 Cytotoxicity and hemolytic assays of CS@PS

Biocompatibility of CS@PS was assessed by cytotoxicity and red blood cell (RBC) hemolytic assays. After FL83B and HepG2 cells were inoculated in 96-well plates for 24 h, 10 μL of CS@PS (diluted to different concentration gradients with complete medium) was added to each well. The cells were incubated for 48 h, 10 μL of CCK8 (Beyotime Biotechnology, Shanghai, China) was added to each well and incubated for another 2 h. Data were collected on a 450 nm multimode enzyme labeler (BioTek Synergy H1). For hemolytic assays, CS@PS in PBS (1, 2 and 4 mg/mL) were co-cultured with 4 % RBCs for 1 h at 37 °C. Deionized water and PBS were used as positive and negative controls. The extent of RBC lysis was determined by measuring the amount of hemoglobin released by spectrophotometry (from 525 to 625 nm).

### 4.6 Cellular uptake and endosomes escape of CS@PS

Cellular uptake was studied by flow cytometry. Briefly, Cy5-labeled CAT and Cy3-labeled SOD were dispersed in PBS buffer, and other procedures were remained the same to obtain Cy5-CAT/Cy3-SOD@PS. FL83B and HepG2 cells were added in a 6-well plate at a density of 2 × 10^4^ cells per well. After 24 h, 20 μL of free Cy5-CAT and Cy3-SOD, and Cy5-CAT/Cy3-SOD@PS were added to each well for 4 h. FL83B and HepG2 cells were harvested and resuspended in 500 μL PBS solution and then analyzed by flow cytometry (Beckman Coulter CytoFLEX S). Endosomal escape was observed on a confocal microscopy platform (CLSM, Leica STELLARIS 5). FL83B and HepG2 cell cells were added at a density of 2 × 10^4^ cells in 35 mm confocal glass-bottomed dishes. After 24 h, 20 μL of CS@PS-DiD were added to each well for 6 h. The cells were then stained with Lysotracker Red/DAPI and imaged through CLSM using a 63 × oil immersion objective.

### 4.7 *In vitro* ROS scavenging and anti-inflammatory capacity of CS@PS

ROS scavenging and anti-inflammatory capacity were tested in FL83B and HepG2 cells. FL83B and HepG2 cells were cultured in DMEM supplemented with 10 % FBS, Pen/Strep, maintained at 37 °C and 5 % CO_2_. After cultured in 12-well plates for 24 h, cells were treated with CS@PS and free CAT/SOD (CAT and SOD dosages: 30 U), and then continued to incubate for 12 h. To study the ROS scavenging ability, H_2_O_2_ (100 μM) was added to each group of cells and incubated for another 12 h. For anti-inflammatory, after cultured in 12-well plates for 24 h, cells were stimulated with LPS (500 ng/mL) for 3 h, following treated with CS@PS and free CAT/SOD for 12 h, respectively. DCFH-DA was added and ROS levels in each group were quantified by fluorescence microscopy and flow cytometry.

### 4.8 *Ex vivo* imaging of CS@PS in the liver

CS@PS was labeled with DiD dye for fluorescence imaging. The distribution of CS@PS in major organs of mice, including the heart, liver, spleen, lungs, and kidneys, was analyzed with IVIS Spectrum imaging system (PerkinElmer, Waltham, MA). CS@PS-DiD was injected through the tail vein (6-8-week-old female C57BL/6 mice, injection dose of 200 μL per mouse), and the mice were executed after 6 h. Mice were dissected to collect organs and DiD channel fluorescence imaging was performed to analyze the biodistribution of CS@PS. Mouse livers were homogenized an passed through a cell sieve to obtain a cell suspension, The suspension was then centrifuged, re-dispersed, lysed to remove erythrocytes, ultimately yielding a single-cell suspension of liver tissue. Different types of cells in mouse liver were labeled with antibodies and incubated at room temperature for 30 min. The dispersed cell suspensions in PBS were detected by flow cytometry.

### 4.9 *In vivo* therapeutic effect of CS@PS against DILI

DILI mice model was established by intraperitoneally injecting with a single dose of acetaminophen (350 mg/kg). After 6 h, the first drug intervention was carried out using CS@PS (CAT and SOD dosages: 1500 U), followed by the following drug intervention at 24 and 48 h, respectively. Blood biochemistry, metabolomics and proteomics were performed during treatment. The liver tissue damage and ROS levels in liver were examined by H&E staining and ROS staining. The blood biochemistry and blood routine of the mice received CS@PS were examined, and pathological sections of the major organs were stained and analyzed to evaluate the *in vivo* biosafety of CS@PS.

### 4.10 Statistical analyses

The data were plotted as mean ± SD and analyzed for statistical significance by one-way ANOVA with Tukey multiple comparisons tests or group *t*-tests using Prism 10. P values ∗p < 0.05 was considered significant, and ∗∗p < 0.01, ∗∗∗p < 0.001 were highly significant.

## CRediT authorship contribution statement

**Wenxing Gu:** Writing – original draft, Methodology, Investigation, Funding acquisition, Formal analysis, Data curation, Conceptualization. **Ruxue Bai:** Investigation, Formal analysis, Data curation. **Jiaxin Wang:** Methodology, Investigation. **Jun Wang:** Investigation. **Chongzhou Fang:** Methodology, Data curation. **Yuanchao Shi:** Investigation, Formal analysis. **Peixing Wang:** Investigation. **Qiaoqiao Wang:** Investigation. **Wei Bing:** Writing – original draft, Supervision. **Tian Xie:** Writing – review & editing, Supervision. **Jing Mu:** Writing – review & editing, Methodology, Funding acquisition, Conceptualization.

## Declaration of competing interest

The authors declare no conflict of interest.

## Data Availability

Data will be made available on request.
