# Supplementary material for: Biomimetic polymersomal nanoreactors for hepatocyte-directed detoxification in drug-induced liver injury
Source: Mater Today Bio. 2025 Aug 5;34:102169. doi: 10.1016/j.mtbio.2025.102169 (PMC12536553; doi:10.1016/j.mtbio.2025.102169)
Supplement: Multimedia component 1 [file mmc1.docx]

**Supporting information**

**Biomimetic Polymersomal Nanoreactors for Hepatocyte-Directed Detoxification in Drug-Induced Liver Injury**

Wenxing Gu^a,b^, Ruxue Bai^a,c^, Jiaxin Wang^a^, Jun Wang^a^, Chongzhou Fang^a^, Yuanchao Shi^a^, Peixing Wang^b^, Qiaoqiao Wang^b^, Wei Bing^c,d^*, Tian Xie^b^*, Jing Mu^a^*

^a^ Institute of Precision Medicine, Peking University Shenzhen Hospital, Shenzhen, 518036, P. R. China

^b^ School of Pharmacy, Zhejiang Provincial Key Laboratory of Anti-Cancer Chinese Medicines and Natural Medicines, Hangzhou Normal University, Hangzhou, 311121, P. R. China

^c^ School of Chemistry and Life Science, Changchun University of Technology, Changchun, 130012, China

^d^ Key Laboratory of Bionic Engineering, Ministry of Education, Jilin University, Changchun 130022, China

﻿*Corresponding authors. E-mail addresses: [jing.mu@pkuszh.com](mailto:jing.mu@pkuszh.com) (Jing Mu)

**Figure S1** Schematic synthesis of polymers PA9 and ZP3.


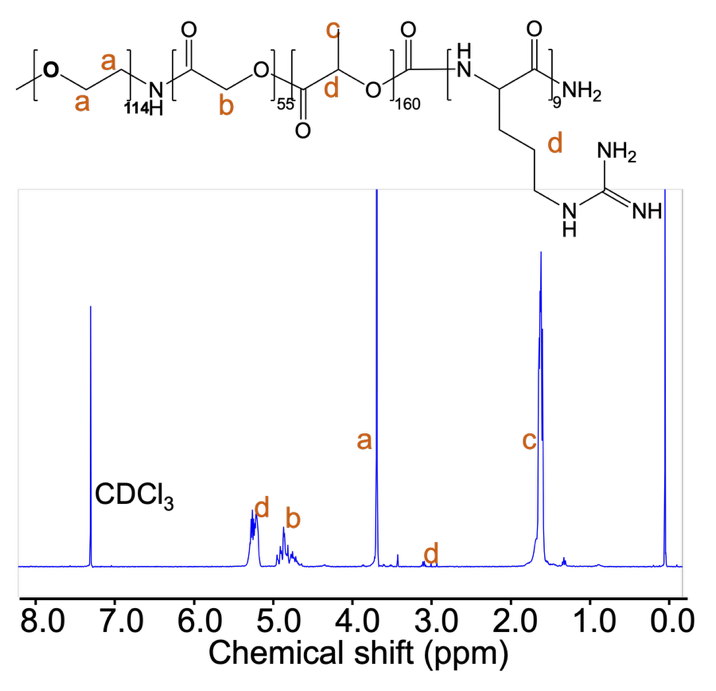


**Figure S2** ^1^H NMR spectrum of polymer PA9.

**Figure S3** ^1^H NMR spectrum of polymer ZP3.

**Figure S4** CAT activity of the CS@PS, CS (CAT and SOD).

**Figure S5** SOD activity of the CS@PS, CS (CAT and SOD).

**Figure S6** O_2_^•−^ elimination rate of the CS@PS, CS (CAT and SOD).


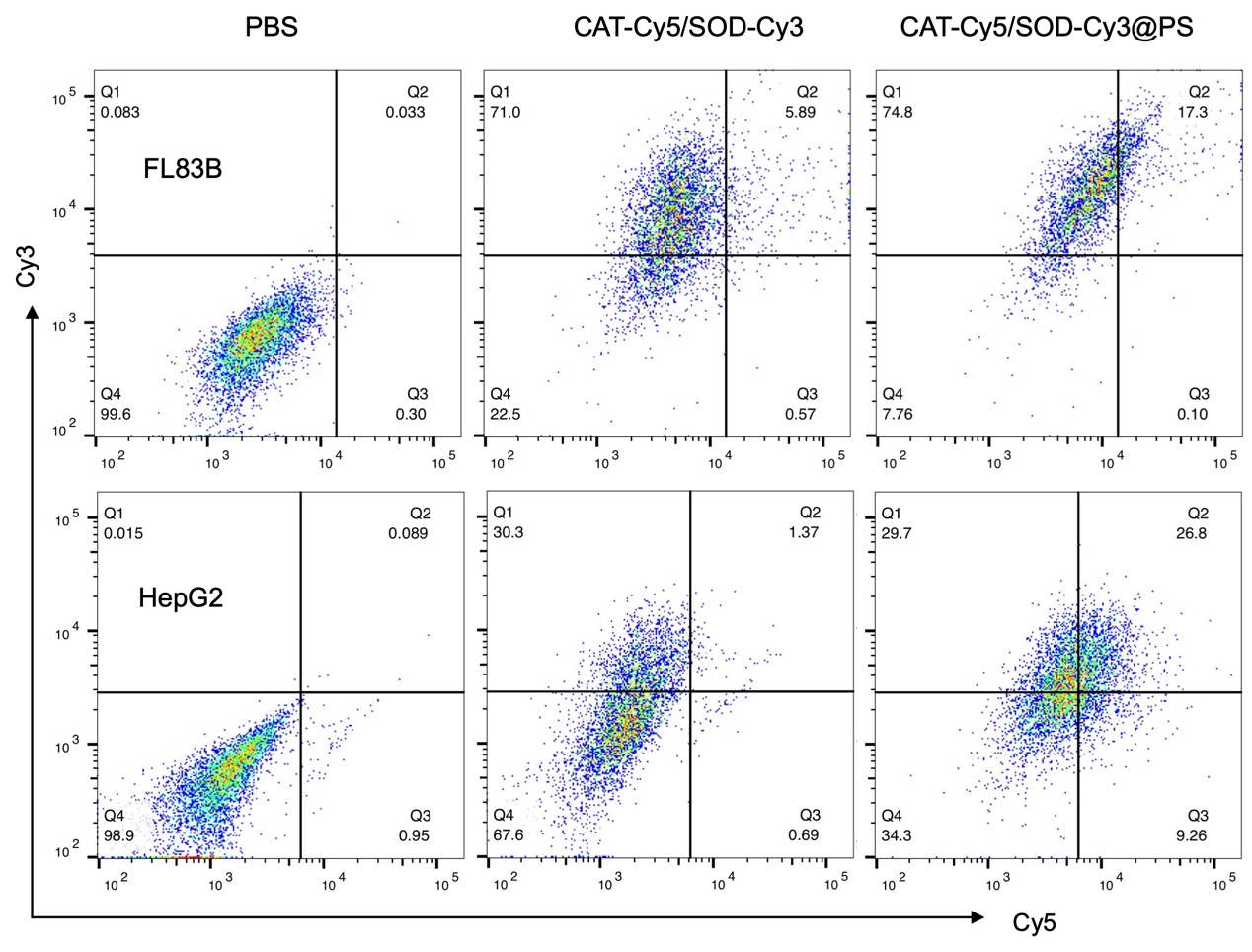


**Figure S7** Cellular uptake efficiency analysis by flow cytometry in FL83B and HepG2 cells treated with free CAT-Cy5 and SOD-Cy3, or CAT-Cy5/SOD-Cy3@PS for 4 hours.


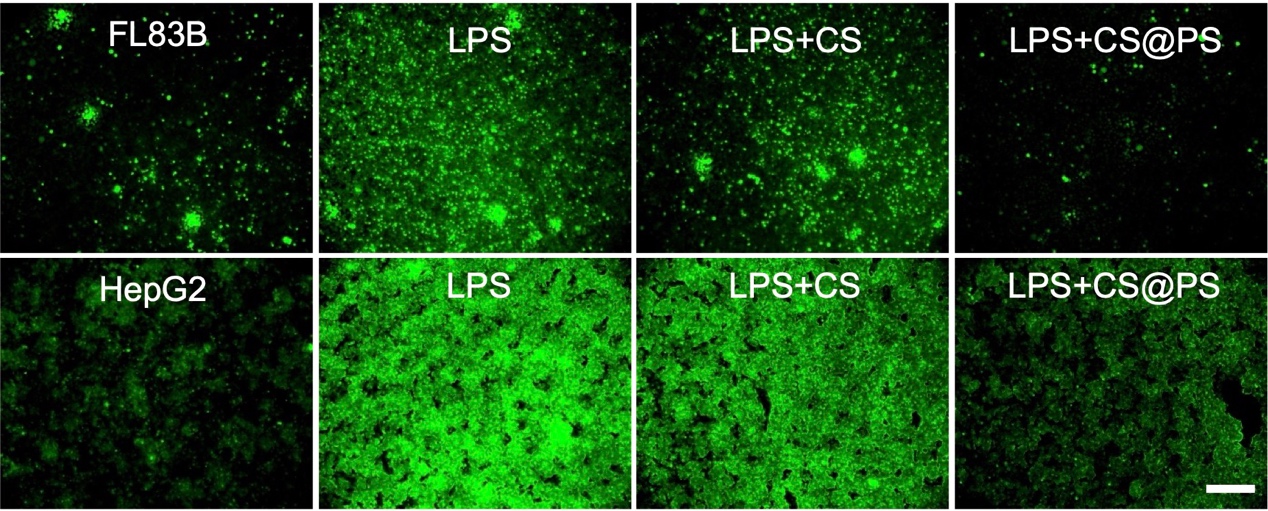


**Figure S8** FL images in untreated, CS (CAT and SOD) and CS@PS-treated FL83B and HepG2 cells stimulated with LPS. Scale bar: 100 μm.


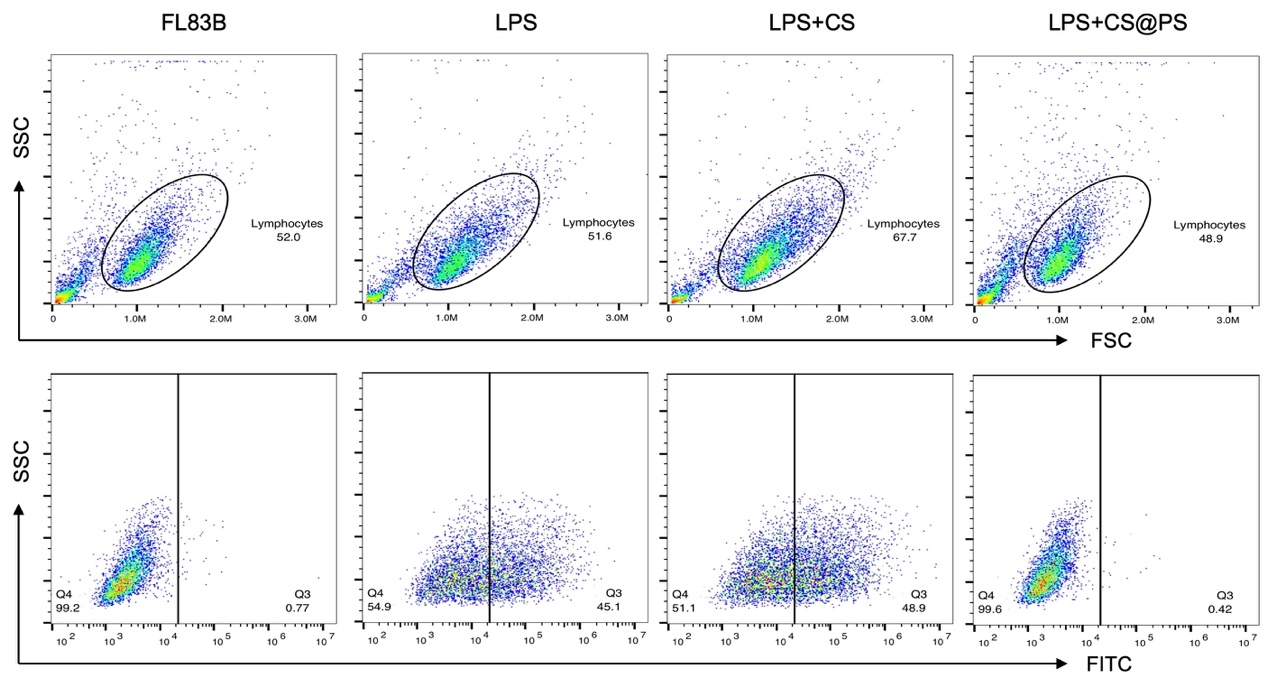


**Figure S9** ROS levels in untreated, CS and CS@PS-treated FL83B cells stimulated with LPS.

**Figure S10** The blood biochemistry and blood routine analysis of mice received CS@PS. Healthy mice as the control.

**Figure S11** ﻿Serum aminotransferase levels (AST and ALT) of mice treated with CS or CS@PS at 78 h (n = 3 mice/group).

**Figure S12** Serum inflammatory factor INF-γ and IL-1β of mice treated with CS or CS@PS at 30 h (n = 3 mice/group).


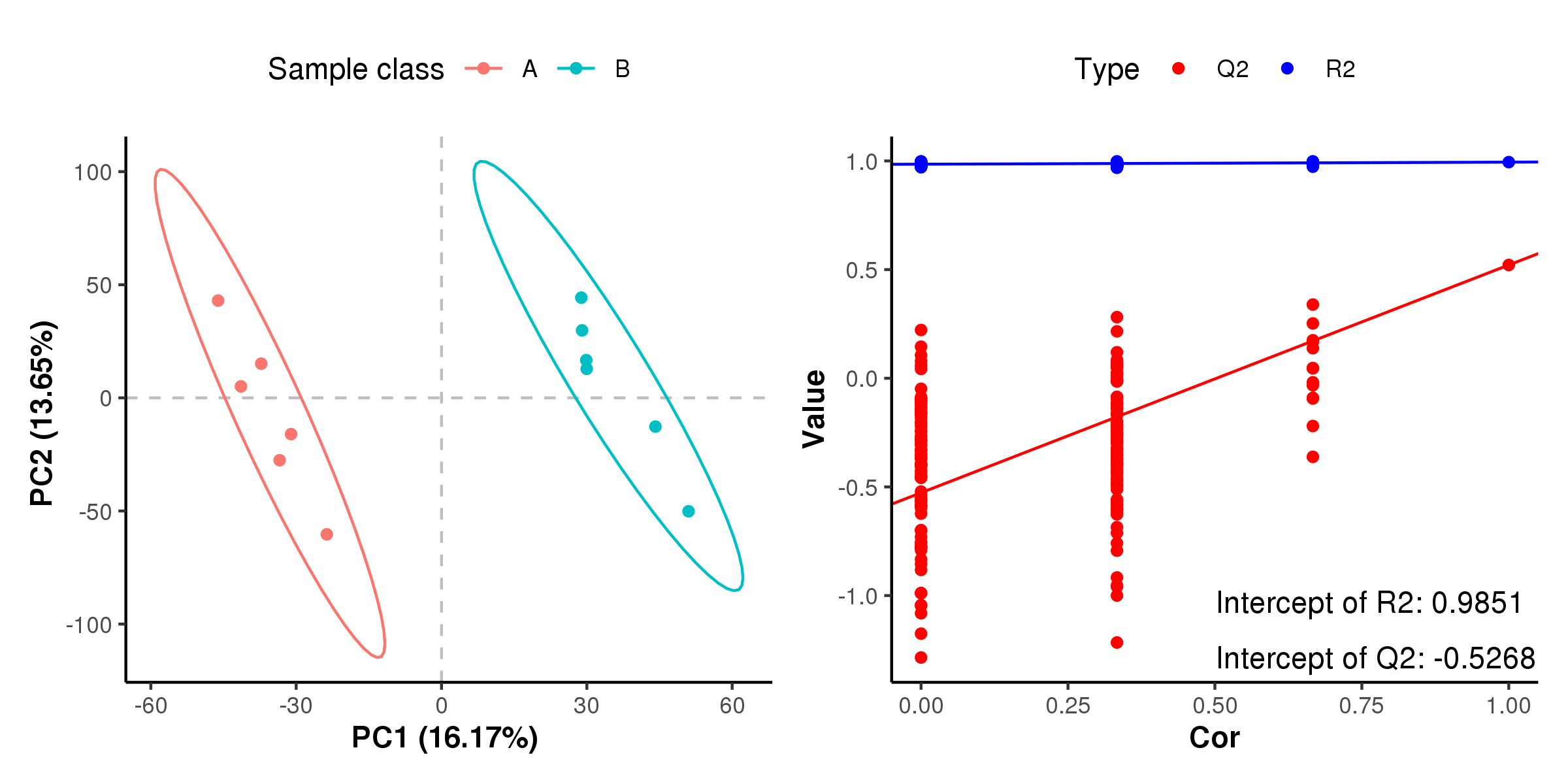


**Figure S13** The PLSDA score plot (left) and the PLSDA model permutation test plot (right) between A and B group. A: Healthy mice, B: APAP group, C: APAP+CS group, D: APAP+CS@PS group.


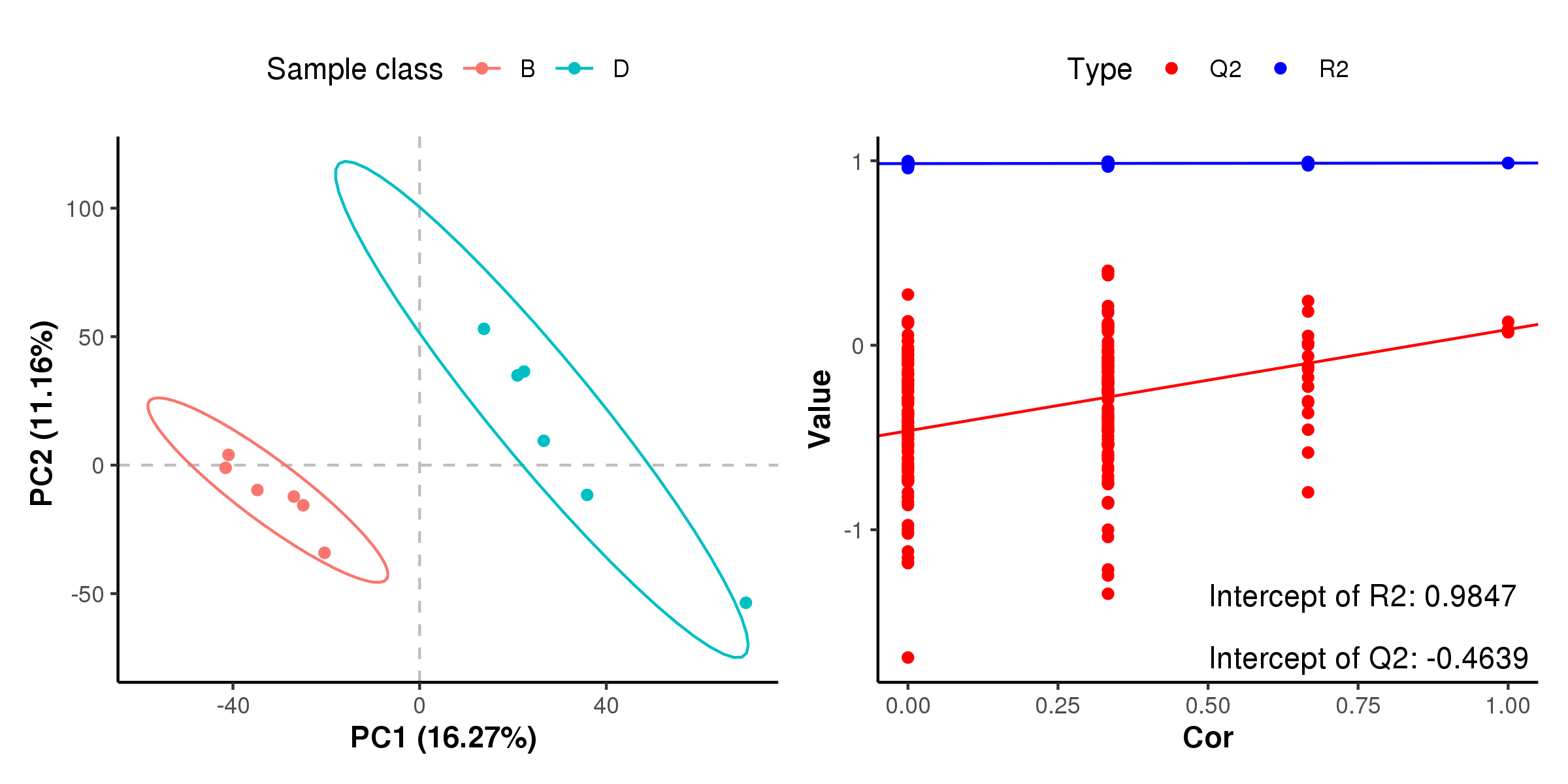


**Figure S14** The PLSDA score plot (left) and the PLSDA model permutation test plot (right) between D and B group. A: Healthy mice, B: APAP group, C: APAP+CS group, D: APAP+CS@PS group.


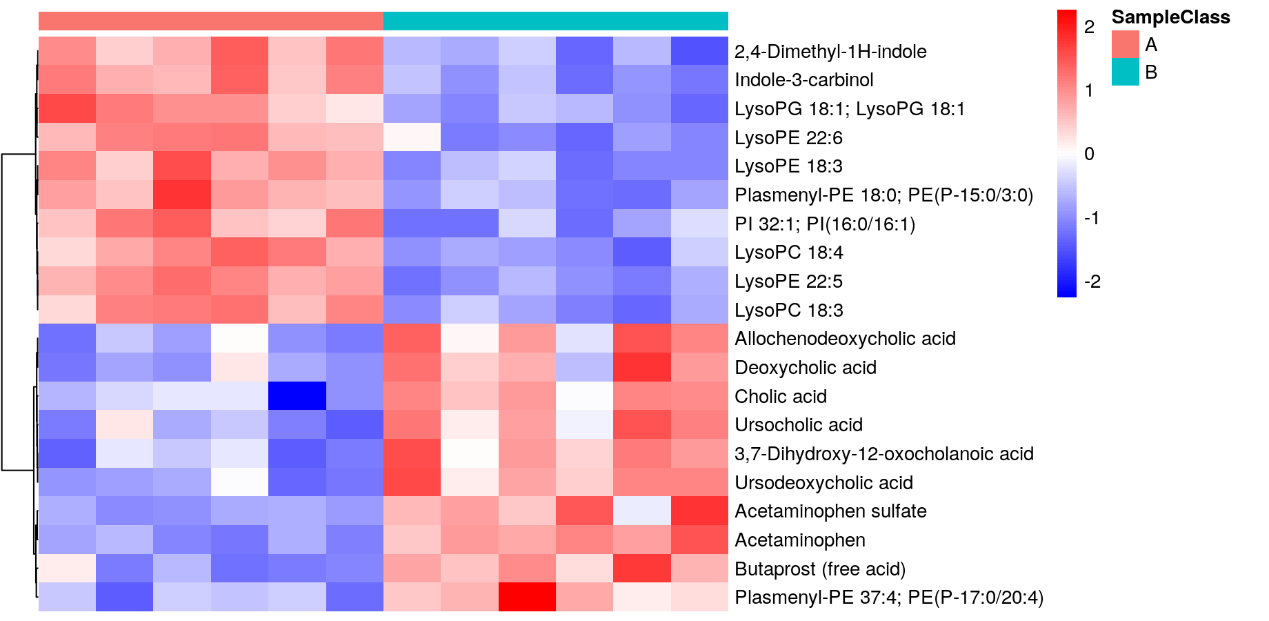


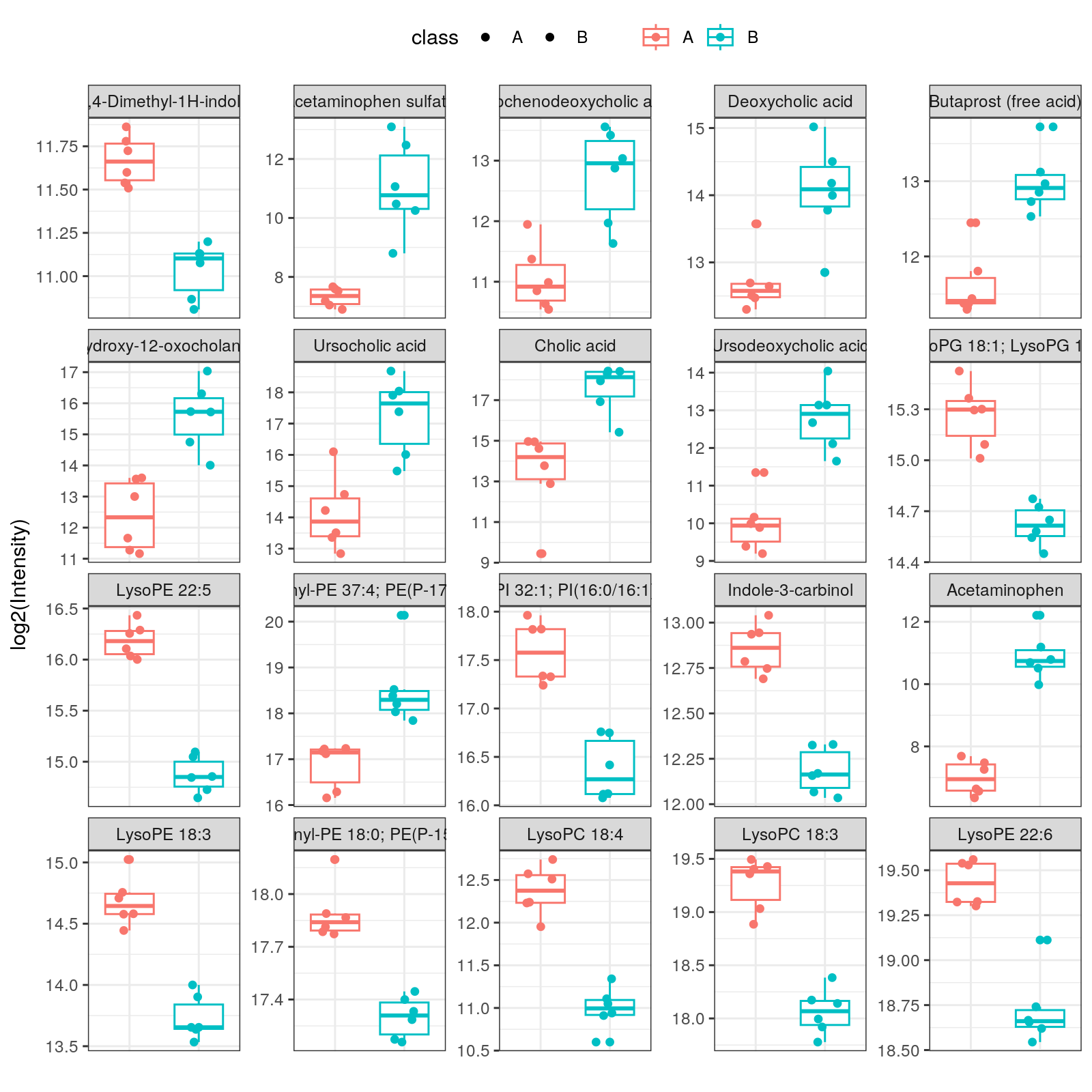


**Figure S15** Thermographic and boxplot analysis of potential biomarkers in the secondary identification of metabolite in B/A comparison.


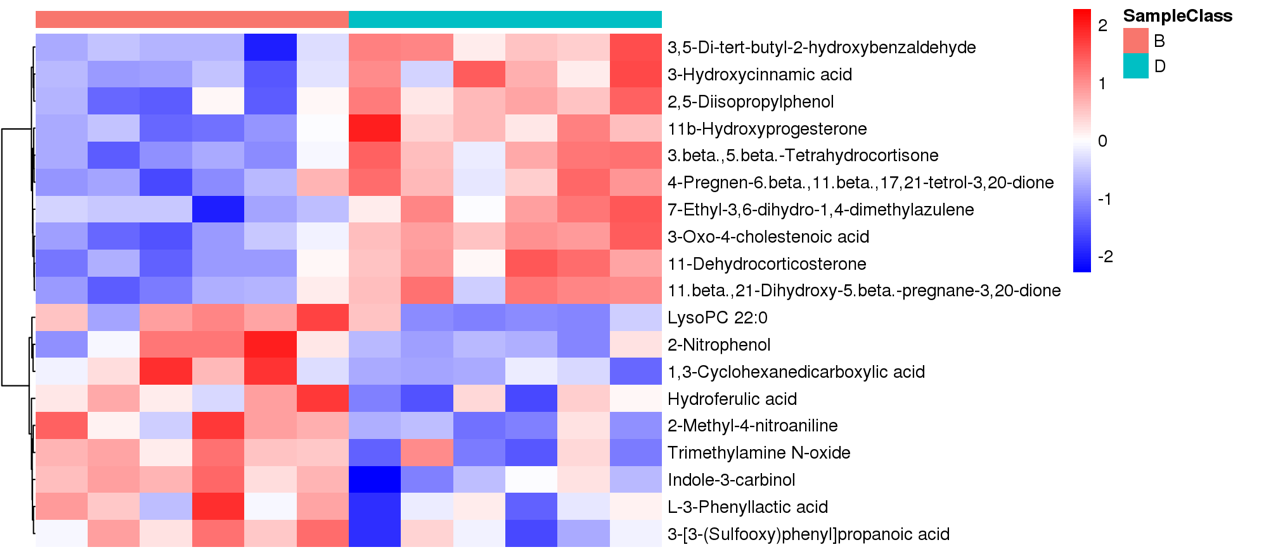


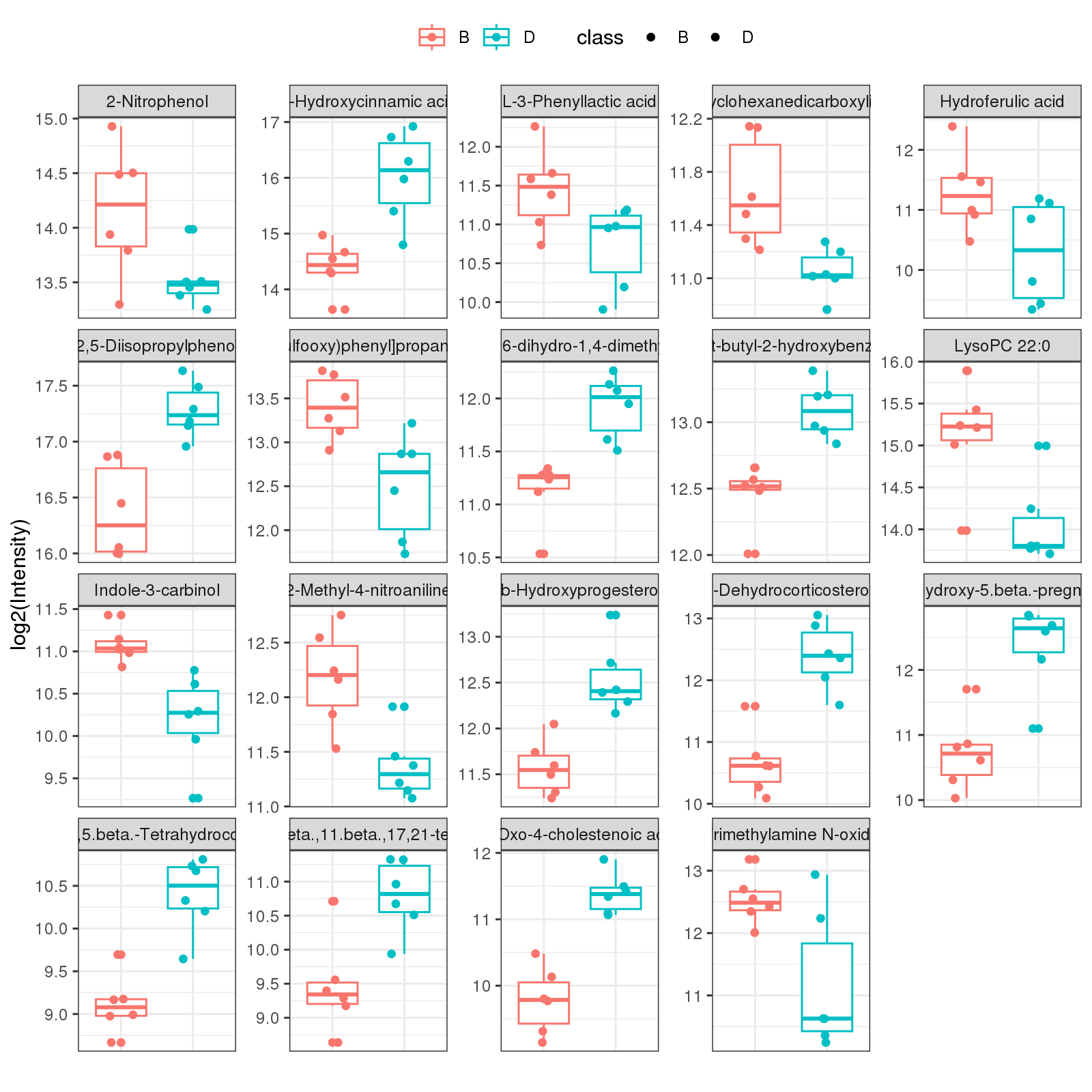


**Figure S16** Thermographic and boxplot analysis of potential biomarkers in the secondary identification of metabolite in D/B comparison.
